# Supplementary material for: Socio-Demographic Factors Influencing Malaria Vaccine Acceptance for Under-Five Children in a Malaria-Endemic Region: A Community-Based Study in the Democratic Republic of Congo
Source: Vaccines (Basel). 2024 Apr 2;12(4):380. doi: 10.3390/vaccines12040380 (PMC11055057; doi:10.3390/vaccines12040380)
Supplement: Supplementary file 1 [file vaccines-12-00380-s001.zip › vaccines-2896364-supplementary.pdf]

Analysis of Vaccine Acceptability and Response to COVID-19, Malaria, and Cholera Vaccination in Bukavu

Objective:
The aim of this study is to evaluate the reactions of local populations to COVID-19 vaccination and to assess the acceptability of other available vaccines as well as those not yet present in our setting. Participation in this study is entirely voluntary, and you may withdraw at any time. Your responses will remain anonymous and confidential.

Ethical Approval:
This study has been approved by the Ethics Committee of the Catholic University of Bukavu, responsible for protecting participant rights. The risks in this study are minimal.

Completion Time:
Responding to this questionnaire will not take more than 5 minutes. If you have any questions related to this research, you can contact the principal investigator, Dr. Patrick Musole Bugeme, via email at musolebugeme@gmail.com or by phone at +243990912809.

I voluntarily agree to answer this questionnaire.
Yes,
No

1. **What is your age (in years)? ***
2. **What is your gender?**
   Male
   Female
3. **What is your religion?**
   Catholic Protestant (ECC, CEPAC, etc.)
   Muslim
   Evangelical Church
   Other
4. **Does your religion accept vaccination? ***
   Yes
   No
   I don't know
5. **In which commune of Bukavu do you reside? ***
   Ibanda commune
   Kadutu commune
   Bagira commune,
   Outside of Bukavu city (specify the region)
6. **I am ***
   Student (from Bachelor's to Master's level)
   Healthcare worker
   None of these options

7. **What is your field of study/faculty?**
   Health sciences (medicine, nursing, laboratory, pharmacy, public health, etc.)
   Other faculties (Economics, Law, Sciences, Social Sciences, etc.)
8. **What is your level of education? ***
   High school diploma
   Bachelor's degree
9. **Have you ever been vaccinated against COVID-19? ***
   Yes
   No
10. **If yes, how many doses of the COVID-19 vaccine have you received?**
    One
    Two
    More than two
11. **If no, why have you not received the COVID-19 vaccine? (Check all that apply)**
    I suspected that the vaccine contained viruses like COVID-19, Ebola, and other microbes
    I feared the side effects
    I thought the vaccine was ineffective
    I thought the vaccine could have other harmful effects
    I didn't know where to get the vaccine I know nothing about the vaccine I refuse to answer this question
12. **If no (not yet vaccinated), I am then willing to receive the COVID-19 vaccine once** available.
    Strongly agree
    Agree
    Neutral
    Disagree
    Strongly disagree
13. **If you have (or would have) children, would you be willing to vaccinate them against COVID-19? ***
    Yes
    No
    I don't know
14. **In COVID-19 vaccination, what type of vaccine do you prefer? ***
    mRNA-based
    DNA-based
    Attenuated virus-based
    Any of them None
15. **COVID-19 is a serious health threat. ***
    Strongly agree
    Agree
    Neutral
    Disagree
    Strongly disagree

16. **COVID-19 can be prevented by vaccination. ***
    Strongly agree
    Agree
    Neutral
    Disagree
    Strongly disagree
17. **The risks of COVID-19 disease are greater than the risks associated with its vaccine. ***
    Strongly agree
    Agree
    Neutral
    Disagree
    Strongly disagree
18. **The COVID-19 vaccines available in our country are safe. ***
    Strongly agree
    Agree
    Neutral
    Disagree
    Strongly disagree
19. **I believe that my government is capable of delivering the COVID-19 vaccine** everywhere in my country, to everyone, and equally. *
    Strongly agree
    Agree
    Neutral
    Disagree
    Strongly disagree
20. **I have confidence in the science that has led to the development of COVID-19** vaccines. *
    Strongly agree
    Agree
    Neutral
    Disagree
    Strongly disagree
21. **I plan to get vaccinated (or revaccinated) against COVID-19 if my employer recommends it to me. ***
    Strongly agree
    Agree
    Neutral
    Disagree
    Strongly disagree
22. **I plan to get vaccinated (or revaccinated) against COVID-19 if my doctor** recommends it to me. *
    Strongly agree
    Agree

Neutral
Disagree
Strongly disagree

23. **I would vaccinate my children against COVID-19 as soon as the vaccine is available to them. ***
    Strongly agree
    Agree
    Neutral
    Disagree
    Strongly disagree
24. **Employers should require COVID-19 vaccination for all their employees. ***
    Strongly agree
    Agree
    Neutral
    Disagree
    Strongly disagree
25. **The government should mandate COVID-19 vaccination for the population. ***
    Strongly agree
    Agree
    Neutral
    Disagree
    Strongly disagree
26. **Universities should require COVID-19 vaccination for all their students. ***
    Strongly agree
    Agree
    Neutral
    Disagree
    Strongly disagree
27. **COVID-19 vaccination should be required for students. ***
    Strongly agree
    Agree
    Neutral
    Disagree
    Strongly disagree
28. **Proof of vaccination should be required for activities in enclosed spaces such as party venues, cinemas, stadiums, etc. ***
    Strongly agree
    Agree
    Neutral
    Disagree
    Strongly disagree
29. **Proof of vaccination should be required for all international travel. ***
    Strongly agree
    Agree

Neutral
Disagree
Strongly disagree

30. **Have you or anyone else in your household experienced a decrease in income due to the COVID-19 pandemic? ***
Yes, a significant decrease
Yes, a moderate decrease
No
31. **Have you or anyone else in your family suffered from COVID-19? ***
Yes
No
32. **Have you lost a family member due to COVID-19? ***
Yes
No
33. **How often in the past week have you felt nervous, anxious, or on edge? ***
Rarely or almost never (less than one day)
Sometimes or a little of the time (1-2 days)
Occasionally (3-4 days)
Most of the time (5-7 days)
34. **In the past week, have you felt down, depressed, or hopeless? ***
Rarely or almost never (less than one day)
Sometimes or a little of the time (1-2 days)
Occasionally (3-4 days)
Most of the time (5-7 days)
35. **Do you believe that the central government will effectively manage various unexpected health threats in our country, including the COVID-19 pandemic? ***
Yes
No
36. **Do you believe that the local government will effectively manage various unexpected health threats in our country, including the COVID-19 pandemic? ***
Yes
No
37. **Have you locally seen an information campaign about COVID-19 vaccination? ***
Yes
No
I am not sure
38. **If yes, where did you come across this campaign? (Check all that apply)**
Online –
on Facebook,
WhatsApp,
and Twitter
I attended an in-person meeting/event
I was visited at home by a COVID-19 vaccination awareness advocate
I heard about it through an announcement about COVID-19 vaccination

I saw it on a poster about COVID-19 vaccination Other:

39. **Have you ever been vaccinated against malaria? ***
    Yes
    No
40. **If no, would you be willing to receive the malaria vaccine once available?**
    Yes
    No
    I don't know
41. **If you have children, would you be willing to have them vaccinated against malaria?**
    Yes
    No
    I don't know
42. **Have you ever been vaccinated against HIV? ***
    Yes
    No

43. **If not, would you be willing to receive the HIV vaccine once available?**
    Yes
    No
    I don't know

44. **If you have children, would you be willing to have them vaccinated against HIV?**
    Yes
    No
    I don't know
45. **Have you ever been vaccinated against tuberculosis? ***
    Yes
    No
46. **If not, would you be willing to receive this vaccine once available?**
    Yes
    No
    I don't know
47. **If you have children, would you be willing to have them vaccinated against tuberculosis?**
    Yes
    No
    I don't know
48. **Have you ever been vaccinated against influenza (flu virus)? ***
    Yes
    No
49. **If not, would you be willing to receive this vaccine once available?**
    Yes
    No

I don't know

50. If you have children, would you be willing to have them vaccinated against the influenza virus?

Yes

No

I don't know

51. Have you ever been vaccinated against cholera? *

Yes

No

52. If not, would you be willing to receive this vaccine once available?

Yes

No

I don't know

53. If you have children, would you be willing to have them vaccinated against cholera?

Yes

No

I don't know

54. Have you ever been vaccinated against HPV (human papillomavirus)? *

Yes

No

55. If not, would you be willing to receive the HPV vaccine once available?

Yes

No

I don't know

56. If you have children, would you be willing to have them vaccinated against HPV?

Yes

No

I don't know

57. Have you ever been vaccinated against hepatitis B virus?

Yes

No

58. If not, would you be willing to receive the hepatitis B virus vaccine once available?

Yes

No

I don't know

59. If you have children, would you be willing to have them vaccinated against the hepatitis B virus?

Yes

No

I don't know

Thank you for your cooperation! You have reached the end of the questionnaire, click "Send"
The research team expresses deep gratitude to you for taking the time and bearing the cost associated with completing this form.

Analyse de l’acceptabilité des vaccins et de la réponse à la vaccination contre la COVID-19, la Malaria et le Cholera à Bukavu

L’objectif de cette étude est d’évaluer les réactions des populations locales face à la vaccination contre la Covid-19 et faire un état de lieu sur l’acceptabilité d'autres vaccins disponibles et ceux non encore présents dans notre milieu. La participation dans cette étude est totalement volontaire et vous pouvez vous retirer à n’importe quel moment. Vos réponses demeureront anonymes et confidentielles.

Cette étude a été approuvée par le Comité d'éthique de l'Université Catholique de Bukavu, en charge de la protection des droits du participant. Les risques dans cette étude sont quasi-minimaux.

Répondre à ce questionnaire ne va pas vous prendre plus de 5 minutes. Si vous avez des questions en rapport à cette recherche, vous pouvez contacter l’investigateur principal, Dr. Patrick Musole Bugeme via: musolebugeme@gmail.com , +243990912809

J'accepte volontairement de répondre à ce questionnaire.
Yes,
No

1. **Quel est votre âge (en année)? ***

2. **Quel est votre genre ?**

Masculin
Féminin

3. **Vous êtes de quelle religion ?**

Catholique
Protestant (ECC, CEPAC,...)
Musulmane
Eglise de reveil
Autres

4. **Est ce que votre religion accepte la vaccination? ***

Oui
Non
Je ne sais pas

5. **Dans quelle commune de Bukavu habitez-vous? ***

commune d'Ibanda
Commune de Kadutu
Bukavu,Commune de Bagira

Hors de la ville de Bukavu
Si, hors de la ville de Bukavu, spécifiez la région dans la quelle vous habitez
province, ville, cité, territoire, chefferie,....

6. **Je suis ***

Etudiant (de Bacc+1 à Bacc+5)
Travailleur dans le domaine de la santé
Aucune de ces deux options

7. **De quelle faculté/domaine d'étude êtes-vous?**

Sciences de la santé (médecine, sciences infirmières, laboratoire, pharmacie, Santé publique,...

Autres facultés que science de la santé (Economie, Droit, faculté des sciences, Sciences sociales,...)

8. **Quel est votre niveau d'étude ? ***

Diplomé d'Etat
Gradué

9. **Avez-vous déjà été vacciné(e) contre la COVID-19? ***

Oui
Non

10. **Si oui, combien de dose de vaccin contre la COVID-19 avez vous déjà reçu?**

Une
Deux
Plus de deux

11. **Si non, selon vous, pourquoi n'avez-vous pas reçu le vaccin contre la COVID-19 ? (cochez toutes les cases qui vous correspondent)**

Je soupçonnais que le vaccin contenait des virus comme le Covid-19, Ebola et d'autres microbes
Je craignais les effets secondaires
Je pensais que le vaccin était innefficace
Je pensais que le vaccin pouvait avoir d'autres effets néfastes
Je ne savais où me procurer ce vaccin
Je ne sais rien du vaccin
Je refuse de répondre à cette question

12. **Si non (non encore vacciné), Je suis alors prêt(e) à recevoir le vaccin contre la COVID-19 une fois disponible?**

Tout à fait d'accord
Un peu d'accord
Je ne suis pas sûr/Je n'ai pas d'opinion
Un peu en désaccord
Pas du tout d'accord

13. **Si vous avez (si vous auriez) des enfants, sériez-vous prêt à les faire vacciner contre la COVID-19? ***

Oui
Non
Je ne sais pas

14. **Dans la vaccination contre la Covid-19, quel type de vaccin préférez-vous ? ***

À base d'ARNm
À base d'ADN
À base des germes attenués
Tous me conviennent
Aucun

15. **La COVID-19 est une menace sanitaire sérieuse ***

Tout à fait d'accord
Un peu d'accord
Je ne suis pas sûr/Je n'ai pas d'opinion
Un peu en désaccord
Pas du tout d'accord

16. **La COVID-19 peut être prévenue par la vaccination ***

Tout à fait d'accord
Un peu d'accord
Je ne suis pas sûr/Je n'ai pas d'opinion
Un peu en désaccord
Pas du tout d'accord

17. **Les risques de la maladie à COVID-19 sont plus grands que les risques liés à son vaccin ***

Tout à fait d'accord
Un peu d'accord
Je ne suis pas sûr/Je n'ai pas d'opinion
Un peu en désaccord
Pas du tout d'accord

18. **Les vaccins contre COVID-19 disponibles chez nous sont sûrs ***

Tout à fait d'accord
Un peu d'accord

Je ne suis pas sûr/Je n’ai pas d’opinion
Un peu en désaccord
Pas du tout d’accord

19. **Je crois que mon gouvernement est capable de délivrer le vaccin contre la COVID-19 partout dans mon pays, à tout le monde et de manière égale ***

Tout à fait d’accord
Un peu d’accord
Je ne suis pas sûr/Je n’ai pas d’opinion
Un peu en désaccord
Pas du tout d’accord

20. **J’ai confiance en la science qui a donné lieu aux vaccins contre la COVID-19. ***

Tout à fait d’accord
Un peu d’accord
Je ne suis pas sûr/Je n’ai pas d’opinion
Un peu en désaccord
Pas du tout d’accord

21. **Je compte me faire vacciner (ou revacciner) contre la COVID-19 si mon employeur me le recommande ***

Tout à fait d’accord
Un peu d’accord
Je ne suis pas sûr /Je n’ai pas d’opinion
Un peu en désaccord
Pas du tout d’accord

22. **Je compte me faire vacciner (ou revacciner) contre la COVID-19 si mon médecin me le recommande ***

Tout à fait d’accord
Un peu d’accord
Je ne suis pas sûr /Je n’ai pas d’opinion
Un peu en désaccord
Pas du tout d’accord

23. **Je ferais vacciner mes enfants contre la COVID-19 dès que ce vaccin sera mis à leur disposition**

Tout à fait d’accord
Un peu d’accord
Je ne suis pas sûr /Je n’ai pas d’opinion
Un peu en désaccord
Pas du tout d’accord

24. **Les employeurs devraient exiger la vaccination contre COVID-19 à tous leurs employés ***

Tout à fait d'accord
Un peu d'accord
Je ne suis pas sûr /Je n'ai pas d'opinion
Un peu en désaccord
Pas du tout d'accord

25. **Le gouvernement devrait exiger à sa population la vaccination contre COVID-19 ***

Tout à fait d'accord
Un peu d'accord
Je ne suis pas sûr /Je n'ai pas d'opinion
Un peu en désaccord
Pas du tout d'accord

26. **Les universités devraient exiger la vaccination contre COVID-19 à tous leurs étudiants ***

Tout à fait d'accord
Un peu d'accord
Je ne suis pas sûr /Je n'ai pas d'opinion
Un peu en désaccord
Pas du tout d'accord

27. **La vaccination contre COVID-19 devrait être exigée aux élèves ***

Tout à fait d'accord
Un peu d'accord
Je ne suis pas sûr /Je n'ai pas d'opinion
Un peu en désaccord
Pas du tout d'accord

28. **Une preuve de vaccination devrait être exigée pour les activités dans les endroits clos comme dans les salles de fête, salles de cinema, les stades… ***

Tout à fait d'accord
Un peu d'accord
Je ne suis pas sûr /Je n'ai pas d'opinion
Un peu en désaccord
Pas du tout d'accord

29. **Une preuve de vaccination devrait être exigée pour tous les voyages à l'étranger ***

Tout à fait d'accord
Un peu d'accord
Je ne suis pas sûr /Je n'ai pas d'opinion
Un peu en désaccord
Pas du tout d'accord

30. **Avez-vous ou quelqu'un d'autre de votre ménage a-t'il demontré une baisse de vos (ses) revenus suite à la pandémie à COVID-19 ? ***

Oui, une baisse grave
Oui, une baisse modérée
Non

31. **Avez-vous ou quelqu'un d'autre dans votre famille souffert de la maladie à COVID-19 ? ***

Oui
Non

32. **Avez-vous perdu un membre de votre famille suite à la maladie à COVID-19 ?**

Oui
Non

33. **Durant la dernière semaine, combien de fois est-ce que vous vous êtes senti nerveux, anxieux ou sur vos nerfs ? ***

Rarement ou Presque pas (moins d'un jour) ;
Quelques fois ou un peu de temps (1-2 jours) ;
Occasionnellement (3-4 jours);
La plupart des temps (5-7 jours)

34. **Durant la semaine passée, vous êtes-vous écroulé, senti dépressif(ve) ou sans espoir ? ***

Rarement ou Presque pas (moins d'un jour) ;
Quelques fois ou un peu de temps (1-2 jours) ;
Occasionnellement (3-4 jours);
La plupart des temps (5-7 jours)

35. **Croyez-vous que le gouvernement central va bien gérer les différentes menaces sanitaires inattendues dans notre pays, incluant la pandémie à COVID-19 ? ***

Oui
Non

36. **Croyez-vous que le gouvernement local va bien gérer les différentes menaces sanitaires inattendues dans notre pays, incluant la pandémie à COVID-19 ? ***

Oui
Non

37. **Avez-vous déjà vu localement une campagne d'information sur la vaccination contre la COVID-19 ? ***

Oui
Non
Je ne suis pas sûr

38. **Si oui, où avez-vous découvert cette campagne ? (Cochez toutes les réponses qui vous correspondent)**

En ligne-sur facebook, Whatsapp et Twitter
J'avais assisté à une réunion/ rencontre en présentiel
J'avais été visité chez moi par un sensibilisateur de la vaccination contre la COVID-19
J'ai entendu cela via une annonce au sujet de la vaccination contre la COVID-19
J'ai vu cela via une affiche au sujet de la vaccination contre la COVID-19
Other:

39. **Avez-vous déjà été vacciné contre la malaria ***

Oui
Non

40. **Si non, sériez-vous prêt à recevoir le vaccin contre la malaria dès que disponible?**

Oui
Non
Je ne sais pas

41. **Si vous avez des enfants, sériez-vous prêt à les faire vacciner contre la malaria?**

Oui
Non
Je ne sais pas

42. **Avez-vous déjà été vacciné contre le VIH ***

Oui

Non

43. **Si non, sériez-vous prêt à recevoir le vaccin contre le VIH dès que disponible?**

Oui
Non
Je ne sais pas

44. **Si vous avez des enfants, sériez-vous prêt à les faire vacciner contre le VIH?**

Oui
Non
Je ne sais pas

45. **Avez-vous déjà été vacciné contre la tuberculose ***

Oui
Non

46. **Si non, sériez-vous prêt à recevoir ce vaccin une fois disponible?**

Oui
Non
Je ne sais pas

47. **Si vous avez des enfants, sériez-vous prêt à les faire vacciner contre la tuberculose?**

Oui
Non
Je ne sais pas

48. **Avez-vous déjà été vacciné contre le virus influenza (virus de la grippe) ***

Oui
Non

49. **Si non, sériez-vous prêt à recevoir ce vaccin dès que disponible?**

Oui
Non
Je ne sais pas

50. **Si vous avez des enfants, sériez-vous prêt à les faire vacciner contre le virus influenza?**

Oui
Non
Je ne sais pas

51. **Avez-vous déjà été vacciné contre le choléra ***

Oui
Non

52. **Si non, sériez-vous prêt à recevoir ce vaccin dès que disponible?**

Oui
Non
Je ne sais pas

53. **Si vous avez des enfants, sériez-vous prêt à les faire vacciner contre le choléra?**

Oui
Non
Je ne sais pas

54. **Avez-vous déjà été vacciné contre le HPV (virus du papillome humain) ***

Oui
Non

Si non, sériez-vous prêt à recevoir le vaccin contre le HPV dès que disponible?
Oui
Non
Je ne sais pas

55. **Si vous avez des enfants, sériez-vous prêt à les faire vacciner contre le HPV?**

Oui
Non
Je ne sais pas

56. **Avez-vous déjà été vacciné contre le virus de hépatite B ***

Oui
Non

57. **Si non, sériez-vous prêt à recevoir le vaccin contre le virus de l'hépatite B dès que disponible?**

Oui
Non
Je ne sais pas

58. **Si vous avez des enfants, sériez-vous prêt à les faire vacciner contre le virus de l'hépatite B?**

Oui
Non
Je ne sais pas

Merci pour votre coopération! Vous êtes à la fin du questionnaire, cliquez sur "Envoyer"
L'équipe de recherche exprime une profonde gratitude envers votre personne pour avoir pris le temps et supporté le coût lié à la complétion de ce formulaire
